# PPARβ/δ Ligands Regulate Oxidative Status and Inflammatory Response in Inflamed Corpus Luteum—An In Vitro Study

**DOI:** 10.3390/ijms24054993

**Published:** 2023-03-05

**Authors:** Karol Mierzejewski, Aleksandra Kurzyńska, Zuzanna Gerwel, Monika Golubska, Robert Stryiński, Iwona Bogacka

**Affiliations:** 1 Department of Animal Anatomy and Physiology, Faculty of Biology and Biotechnology, University of Warmia and Mazury in Olsztyn, 10-719 Olsztyn, Poland; 2 Department of Biochemistry, Faculty of Biology and Biotechnology, University of Warmia and Mazury in Olsztyn, 10-719 Olsztyn, Poland

**Keywords:** corpus luteum, pig, inflammation, oxidative stress, GW0724

## Abstract

Inflammation in the female reproductive system causes serious health problems including infertility. The aim of this study was to determine the in vitro effects of peroxisome proliferator-activated receptor-beta/delta (PPARβ/δ) ligands on the transcriptomic profile of the lipopolysaccharide (LPS)-stimulated pig corpus luteum (CL) in the mid-luteal phase of the estrous cycle using RNA-seq technology. The CL slices were incubated in the presence of LPS or in combination with LPS and the PPARβ/δ agonist—GW0724 (1 μmol/L or 10 μmol/L) or the antagonist—GSK3787 (25 μmol/L). We identified 117 differentially expressed genes after treatment with LPS; 102 and 97 differentially expressed genes after treatment, respectively, with the PPARβ/δ agonist at a concentration of 1 μmol/L or 10 μmol/L, as well as 88 after the treatment with the PPARβ/δ antagonist. In addition, biochemical analyses of oxidative status were performed (total antioxidant capacity and activity of peroxidase, catalase, superoxide dismutase, and glutathione S-transferase). This study revealed that PPARβ/δ agonists regulate genes involved in the inflammatory response in a dose-dependent manner. The results indicate that the lower dose of GW0724 showed an anti-inflammatory character, while the higher dose seems to be pro-inflammatory. We propose that GW0724 should be considered for further research to alleviate chronic inflammation (at the lower dose) or to support the natural immune response against pathogens (at the higher dose) in the inflamed corpus luteum.

## 1. Introduction

Increasing infertility due to chronic inflammation has become a serious problem and a challenge for human and veterinary medicine in recent years. Inflammation is a protective response to pathological conditions such as bacterial infections. However, if the inflammatory cascade is not stopped, it transforms into chronic inflammation and leads to organ dysfunction [1]. An inflammatory response in the female reproductive system is often associated with the presence of lipopolysaccharide (LPS), the endotoxin of Gram-negative bacteria, e.g., *Escherichia coli* (*E. coli*) [2]. LPS binds to TLR and stimulates the synthesis of various pro-inflammatory cytokines such as IL-1β, IL-6, IL-8 and TNF-α [3].

There is evidence that *E. coli* LPS causes infertility by interfering with ovarian follicular development and the ovulation process [4]. Luttgenau et al. [5] reported that luteal TLR2 and TLR4 appear to be involved in the immune response of the corpus luteum (CL), which may be related to the production of pro-inflammatory cytokines and decreased ovarian steroidogenesis in cows. LPS has been reported to alter ovarian axis hormone secretion by affecting GnRH and LH production, CL growth and functions, the timing of ovulation and the estrous cycle [6,7]. In addition, the treatment of cows with LPS altered the structure of the CL and decreased plasma progesterone levels (P_4_), resulting in a temporary suppression of luteal function [8]. Despite these reports, there is a lack of data on the effect of LPS on the functions of the porcine CL. Furthermore, the great anatomical and physiological similarity of the female and porcine reproductive systems and the course of bacterial infection makes the pig a good model for studying the in vitro effects of infection on the immune response in the CL [9].

Peroxisome proliferator-activated receptors (PPARs) are ligand-dependent transcription factors belonging to the nuclear receptor superfamily. To date, three isoforms of PPARs—α, β/δ and γ—have been described [10]. PPARs have been reported to be involved in the various processes necessary for the proper functioning of the ovaries, such as the regulation of steroidogenesis, angiogenesis, tissue remodeling, cell cycle and apoptosis [11]. There is evidence that PPARγ ligands may play a luteotropic role by increasing the activity of 3β-HSD and the secretion of progesterone [12,13]. However, there is limited information on the role of PPARβ/δ ligands in CL function. The anti-inflammatory effects of PPAR ligands have been widely reported, including in our previous work, but most of this relates to the PPARγ isoform [14,15,16]. The effect of PPARβ/δ on inflammation has not been fully elucidated [17]. In some cases, PPARβ/δ agonists appear to exert anti-inflammatory effects, such as inhibiting the synthesis of pro-inflammatory cytokines TNF-α and MCP-1 in the liver, alleviating inflammation in experimental autoimmune encephalomyelitis, or inhibiting diabetic nephropathy by reducing inflammatory mediators in mice [18,19,20]. There are also reports suggesting that PPARβ/δ signaling promotes inflammation [17]. It has been reported that in mice with arthritis, mesenchymal stem cells (MSCs) had higher anti-inflammatory potential than the MSCs derived from PPARβ/δ knockout mice [21].

The present study was conducted to determine the influence of PPARβ/δ ligands on the global transcriptomic profile of the LPS-stimulated corpus luteum of pigs during the mid-luteal phase of the estrous cycle. In addition, transcriptomic changes in the CL after the treatment with LPS alone have been described. For the first time, our research has revealed the role of PPARβ/δ in the regulation of oxidative stress and genes involved in the inflammatory response. In addition, we have shown a dose-dependent effect of the tested agonists. 

## 2. Results

### 2.1. Statistics of RNA Sequencing

**Figure 1:**
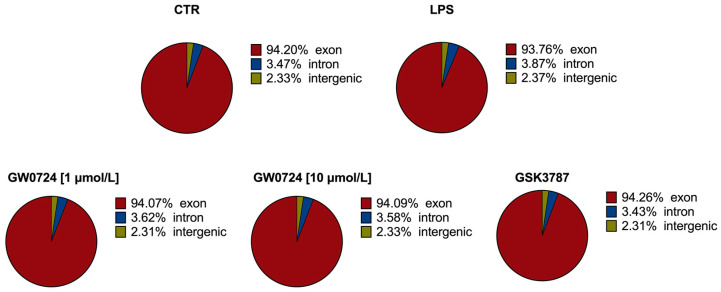
Distribution of mapped reads to genes structures.

RNA sequencing data were created for 20 cDNA libraries, including four untreated samples (controls), four with LPS, four with GW0724 at a concentration of 1 μmol/L, four with GW0724 at a concentration of 10 μmol/L and four with GSK3787. The analysis produced 968,505,414 raw paired-end reads in total, with an average 48,425,271 per sample and a Q20 value that was on average 99.94%. The short reads, low-quality sequences and ambiguous nucleotides were removed from the raw reads, leaving on average of 938,720,608 valid reads per sample, that were used for further analysis (Supplemental Table S2). The filtered reads were mapped to the Ss11.1.98 version of the pig genome with a unique mapped average rate of 94%. The analysis of the distribution of mapped reads to gene structures indicated that 94.11% of read pairs (in average per sample) mapped to coding sequences, 3.56% mapped to introns, and 2.33% mapped to intergenic regions (Figure 1). RNA-seq data have been deposited in the ArrayExpress database at EMBL-EBI under accession number E-MTAB-12027.

### 2.2. The Effect of LPS on Differential Gene Expression in the Corpus Luteum

**Figure 2:**
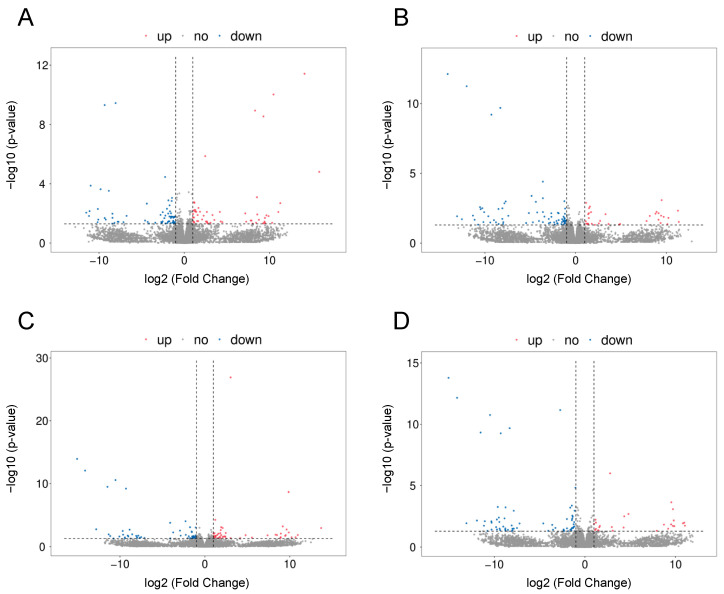
Volcano plots describing the abundance of transcript expression profiles after LPS (**A**); or LPS in combination with: GW0724 at a concentration of 1 μmol/L (**B**), GW0724 at a concentration of 10 μmol/L (**C**), and GSK3787 (**D**) treatment in mid-luteal phase in the corpus luteum. Logarithmic fold changes in expression (log_2_FC) are plotted on the *x*-axis against normalized adjusted *p*-values (*y*-axis). The sharp horizontal line denotes the negative logarithmic adjusted *p*-value (0.05) cut-off. Sharp vertical lines denote the fold change cut-off (absolute value of log_2_FC > 1). Points represent gene expression values, where blue (underexpressed) and red (overexpressed) points denote significant genes (adjusted *p*-value < 0.05). Grey dots indicate non-significant genes.

**Figure 3:**
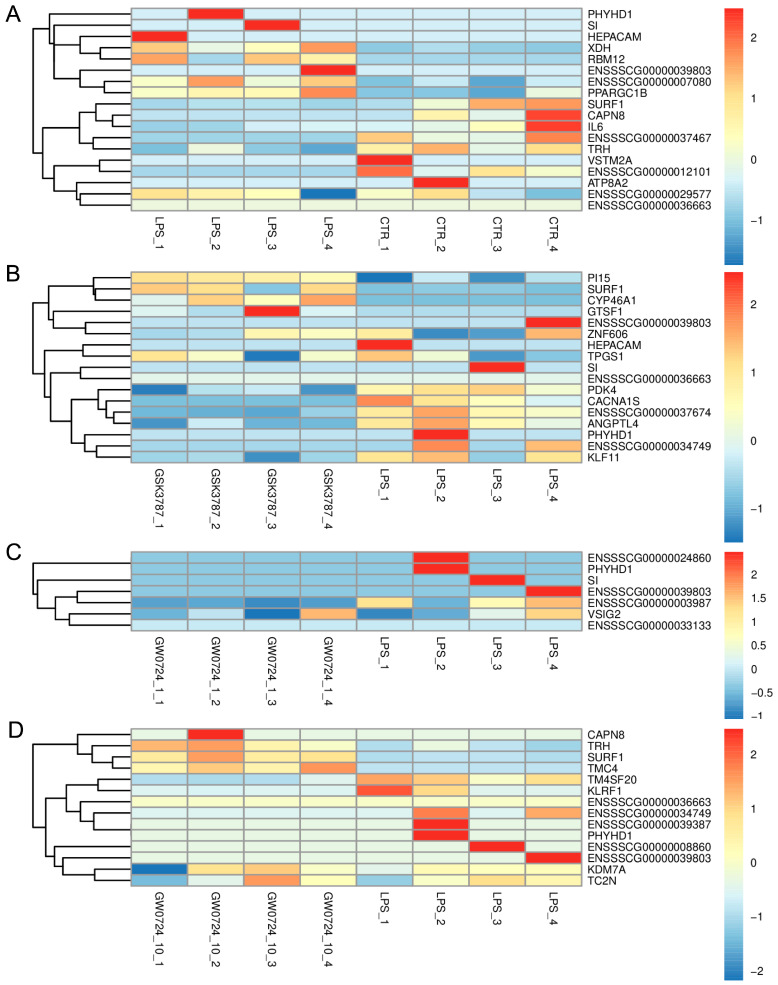
Hierarchical clustering heatmap of differentially expressed genes after LPS (**A**), GW0724 at a concentration of 1 μmol/L (**B**), GW0724 at a concentration of 10 μmol/L (**C**), or GSK3787 (**D**) treatment of porcine corpus luteum in the late-luteal phase of the estrous cycle.

**Figure 4:**
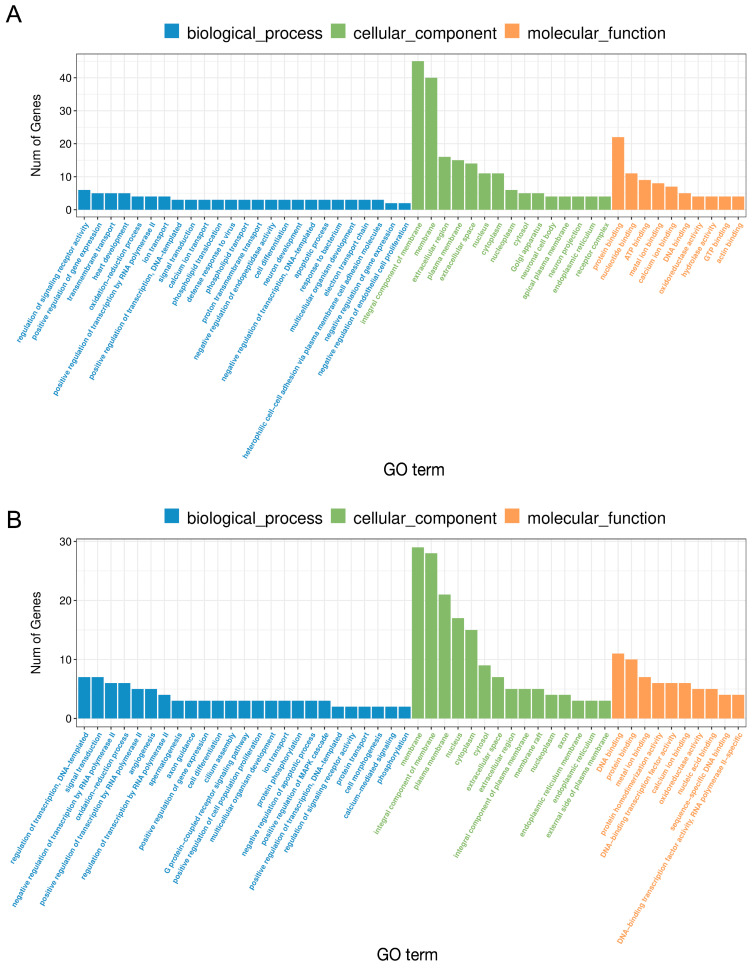
HierarchGO analysis of the differentially expressed genes after the treatment of the corpus luteum with LPS (**A**) or GW0724 at a concentration of 1 μmol/L (**B**) in the mid-luteal phase of the estrous cycle. The *x*-axis indicates the terms of biological processes, while the *y*-axis indicates the number of significant enriched genes (*p* < 0.05).

The RNA-Seq analysis revealed 117 DEGs (63 downregulated and 54 upregulated) in porcine CL on days 10–12 after LPS treatment (Figure 2A and Figure 3A). The Gene Ontology (GO) analysis assigned these DEGs to 159 terms of biological processes, 18 terms of cellular components, and 47 terms of molecular functions (Figure 4A). The treatment of the CL tissue with LPS altered the expression of genes involved in processes such as the regulation of signaling receptor activity (*INSL6*, *IL-6*, *TNFSF14*, *IFN-DELTA-7*, *PDYN*, *PRL*), response to bacterium (*C15orf48*, *NLRP6*, *ENSSSCG00000037358*) or oxidoreductase activity (*ALOX12B*, *ALDH3B2*, *XDH*, *SOD2*). Moreover, KEEG enrichment analysis revealed that DEGs were involved in signaling pathways such as cytokine–cytokine receptor interaction (*TNFRSF9*, *IL-6*, *TNFSF14*, *IL-27*, *PRL*) or the NOD-like receptor signaling pathway (*NLRP6*, *IL-6*, *ENSSSCG00000007964*) (Supplemental Figure S1A). All detailed DEGs, GO and KEEG results were described in Supplemental Tables S3–S5, respectively.

### 2.3. The Effect of PPARβ/δ Agonist on Differential Gene Expression in the Corpus Luteum

The results of our study showed that the PPARβ/δ agonist GW0724 at a concentration of 1 μmol/L affected the expression of 102 protein-coding genes (74 downregulated and 28 upregulated) (Figure 2B and Figure 3B). The GO analysis assigned these DEGs to 193 terms of biological processes, 13 terms of cellular components, and 37 terms of molecular functions (Figure 4B). These DEGs were involved, for example, in oxidation–reduction processes (*CYP46A1*, *CYP4A24*, *ALOX12B*, *ALDH3B2*), immune response (*IL-15*, *CSF3*, *TNFSF14*, *VTN*) or cell population proliferation (*SHH*, *MAB21L2*, *CSF3*). Furthermore, KEEG analysis showed that these DEGs were engaged in pathways such as cytokine–cytokine receptor interaction (*CD27*, *IL-15*, *CSF3*, *TNFSF14*) or drug metabolism (*TK1*, *ENSSSCG00000040980*, *ALDH3B2*) (Supplemental Figure S1B). All detailed DEGs, GO and KEEG results were described in Supplemental Tables S6–S8 respectively. 

**Figure 5:**
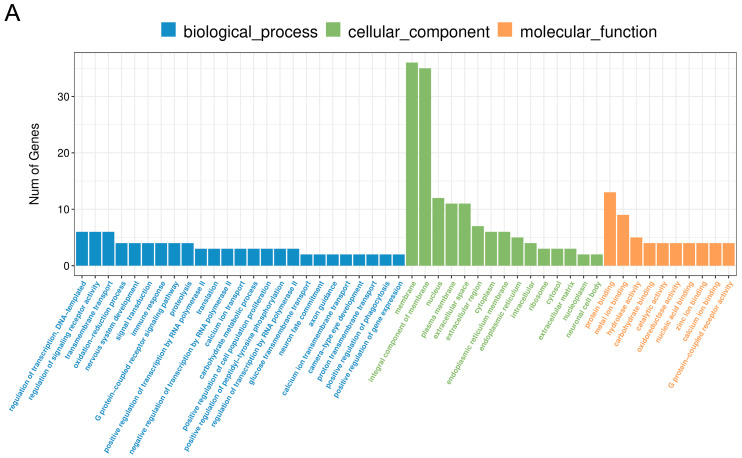

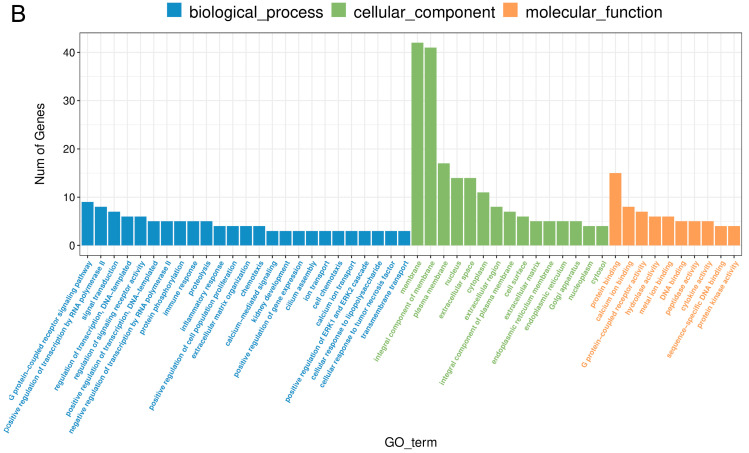
GO analysis of the differentially expressed genes after the treatment of the corpus luteum with GW0724 at a concentration of 10 μmol/L (**A**) or GSK3787 (**B**) in the late-luteal phase of the estrous cycle. The *x*-axis indicates the terms of biological processes, while the *y*-axis indicates the number of significant enriched genes (*p* < 0.05).

In turn, the treatment of the CL with PPARβ/δ agonist GW0724 at a concentration of 10 μmol/L resulted in changes in the expression of 103 genes (57 downregulated and 46 upregulated) (Figure 2C and Figure 3C). The GO analysis assigned these DEGs to 275 terms of biological processes, 18 terms of cellular components, and 42 terms of molecular functions (Figure 5A). These DEGs were involved, for example, in immune and inflammatory response (*LTA*, *CCL3L1*, *IL-6*, *TNFSF14*, *CCL4*, *ELF3*), chemotaxis (*PDGFRA*, *CCL3L1*, *ENSSSCG00000020934*, *CCL4*), cellular response to lipopolysaccharide (*CD180*, *IL-6*, *ZFP36*) and tumor necrosis factor (*CCL3L1*, *ZFP36*, *CCL4*), or cytokine activity (*LTA*, *CCL3L1*, *IL-6*, *TNFSF14*, *CCL4*). Additionally, KEEG analysis indicated that these DEGs were engaged in pathways such as the NF-kappa B signaling pathway (*LTA*, *TNFSF14*, *CCL4*) or Toll-like receptor signaling pathway (*LTA*, *CCL3L1*, *IL-6*, *CCL4*) (Supplemental Figure S1C). All detailed DEGs, GO and KEEG results were described in Supplemental Tables S9–S11, respectively.

### 2.4. Comparative Analysis between Two Doses of PPARβ/δ Agonist

Statistical analysis identified 97 DEGs (19 downregulated and 78 upregulated) in the CL treated with GW0724 at a concentration of 10 μmol/L compared with 1 μmol/L (Supplemental Figure S2A, Supplemental Figure S2B). The GO analysis assigned these DEGs to 148 terms of biological processes, 8 terms of cellular components, and 32 terms of molecular functions (Supplemental Figure S2D). These DEGs were involved, for example, in immune and inflammatory response (*ENSSSCG00000007642*, *CCL19*, *CSF3*, *MBL1*, *IL17B*, *CCR3*), oxidation–reduction processes (*CYP4A24*, *HSD17B3*, *SURF1*) or response to DNA damage stimulus (*MRNIP*, *BATF*). Moreover, KEEG analysis indicated that these DEGs were engaged in pathways such as cytokine–cytokine receptor interaction (*CCL19*, *IL17B*, *CSF3*, *CCR3*, *IL-27*) and the IL-17 signaling pathway (*IL17B*, *CSF3*) (Supplemental Figure S2C). All detailed DEGs, GO and KEEG results were described in Supplemental Tables S12–S14, respectively. 

### 2.5. The Effect of PPARβ/δ Antagonist on Differential Gene Expression in the Corpus Luteum

The study demonstrated that PPARβ/δ antagonist GSK3787 affected the expression of 88 protein-coding genes (63 downregulated and 25 upregulated) (Figure 2D and Figure 3D). The GO analysis assigned these DEGs to 250 terms of biological processes, 16 terms of cellular components, and 39 terms of molecular functions (Figure 5B). These DEGs were mostly assigned to oxidation–reduction processes and oxidoreductase activity (*CYP46A1*, *ENSSSCG00000003963*, *ALOX12B*, *ENOX1*, *CRYZL1*, *ENSSSCG00000030195*) as well as angiogenesis (*ANGPTL4*, *SHH*, *EPHB1*, *HAND1*, *LEP*). Moreover, KEEG analysis indicated that these DEGs were engaged in pathways such as the PPAR signaling pathway (*ANGPTL4*, *PLIN2*) and cAMP signaling pathway (*GRIN2B*, *GHRL*, *CACNA1S*, *PLN*) (Supplemental Figure S1D). All detailed DEGs, GO and KEEG results were described in Supplemental Tables S15–S17, respectively.

### 2.6. Real-Time PCR Analysis

The treatment of the CL with LPS increased PPARβ/δ mRNA abundance during the mid-luteal phase of the estrous cycle (Supplemental Figure S3). Real-time PCR expression patterns of the tested DEGs (*IL-6*, *SOD2*, *CD180*, *ANGTPL4*) were in agreement with RNA-Seq results (Supplemental Figure S4). 

### 2.7. Biochemical Analyses

**Figure 6:**
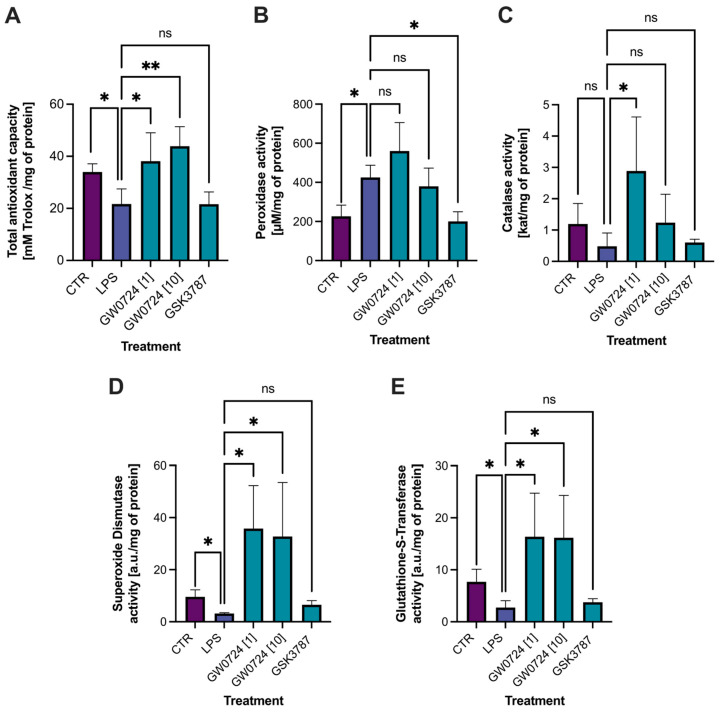
Different biochemical indicators of oxidative stress in corpus luteum treated with LPS or with LPS and PPARβ/δ agonist (GW0724 at a concentration of 1 μmol/L or 10 μmol/L) and antagonist (GSK3787). Visualization of results of performed biochemical analyses: total antioxidant capacity (**A**), peroxidase activity (**B**), catalase activity (**C**), superoxidate dismutase activity (**D**), glutathione-S-transferase activity (**E**). Results were considered statistically significant at *p* ≤ 0.05 (*) and *p* ≤ 0.002 (**); ns: not significant.

Total antioxidant capacity was lower in the LPS-treated CL (21.66 mM Trolox/mg protein) compared with the control (33.97 mM Trolox/mg protein). Analysis of the CL, treated with PPARβ/δ agonist at a concentrations of 1 μmol/L and 10 μmol/L, showed higher TAC levels compared with the LPS-treated CL. Moreover, TAC was enhanced with increasing agonist concentration (38.1 and 43.9 mM Trolox/mg protein, respectively). The total antioxidant capacity of the CL treated with the antagonist was similar to that of the CL treated with LPS (21.63 mM Trolox/mg protein) and no statistical difference was noted (Figure 6A).

The activity of peroxidase in the CL increased almost 2-fold after stimulation with LPS compared with the control (226.4 vs. 424.8 μM/mg protein). The difference in peroxidase activity in the agonist-treated CL compared with LPS-treated CL was not statistically significant. However, peroxidase activity in CL, which was treated with an antagonist (200.2 μM/mg protein), was decreased almost two-fold compared with LPS-treated CL (424.8 μM/mg protein) (Figure 6B).

The activity of catalase in the CL did not change after LPS administration. Only the lower concentration of agonist compared with LPS-treated CL increased catalase activity (0.47 vs. 2.88 kat/mg protein) (Figure 6C).

The trend of the activity of SOD and GST was similar. The treatment of the CL with LPS decreased the activity of SOD almost three-fold compared with the control (9.58 vs. 3.18 a.u./mg protein). In turn, PPARβ/δ agonist GW0724 at concentrations of 1 μmol/L or 10 μmol/L increased the activity of SOD compared with the LPS-treated CL (1 μmol/L–35.77 and 10 μmol/L–32.72 a.u./mg protein) (Figure 6D). A similar observation was made with respect to GST activity. The treatment of the CL with LPS decreased the activity of GST compared with the control (7.69 vs. 2.74 a.u./mg protein), while the treatment with the agonist increased the activity of GST at both low and high concentrations (16.37 and 16.16 a.u./mg protein, respectively) compared with the LPS-treated CL. The activity of SOD or GST was not significantly affected by the PPARβ/δ antagonist (Figure 6E).

## 3. Discussion

A growing body of evidence shows a negative impact of lipopolysaccharide from *Escherichia coli* on reproductive functions. There are reports indicating that LPS leads to infertility by impairing ovarian functions [4]. It has been shown that LPS accumulates in follicular fluid, decreases the production of estradiol from granulosa cells, suppresses the expression of gonadotrophin receptors and disrupts blastocyst implantation [22]. Despite this evidence, transcriptome changes in the porcine corpus luteum under the influence of LPS had never been studied. The present results demonstrate for the first time the global transcriptomic profile of the CL of gilts during the mid-luteal phase of the estrous cycle and the effect of LPS as well as PPARβ/δ ligands during LPS-induced inflammation within the structure. We demonstrated that LPS affected the expression of 118 DEGs (63 of which were downregulated, whereas 55 were upregulated). These DEGs were assigned to different biological processes, such as response to bacterium, the negative regulation of endothelial cell proliferation, or the IL-17 signaling pathway.

Among the above genes with altered expression after LPS stimulation, we identified those involved in the regulation of oxidative stress and reactive oxygen species (ROS) production (*XDH*, *ALDH3B2*, *SOD2*, *ALOX12B*). It has been frequently reported that ROS play a significant and diverse role within the ovary, especially in the CL during luteal regression [23]. In addition, the abruptly increased production of ROS (e.g., by LPS during bacterial infection) decreases P_4_ secretion, which may contribute to functional and structural luteolysis and disturb the proper course of the estrous cycle [24]. Xanthine dehydrogenase (XDH) is the rate-limiting enzyme for purine degradation, metabolizing hypoxanthine/xanthine to uric acid [25]. During these metabolic processes, numerous ROS are produced, including superoxide anion (O_2_^•−^) and hydrogen peroxide (H_2_O_2_) [26]. In the present study, we demonstrated that *XDH* was upregulated in the LPS-treated CL during the mid-luteal phase of the estrous cycle. Moreover, we found that LPS downregulated the expression of *ALDH3B2* in the CL. This gene belongs to the aldehyde dehydrogenase (ALDH) family of enzymes, which is critical for the detoxification of aldehydes [27]. ALDH3B1 has been reported to metabolize and protect cells from aldehydes and oxidative compounds derived from lipid peroxidation (LPO), suggesting an important role of this enzyme in cellular defense against oxidative stress and downstream aldehydes [28]. Mishra et al. [29] reported that the exposure of bovine luteal cells to LPS increased the LPO process. Based on our results, we can assume that LPS intensifies LPO and oxidative stress by increasing the expression of *XDH* and decreasing *ALDH3B2* in the porcine CL. Our studies revealed also that LPS increased the expression of *SOD2* in the CL during the mid-luteal phase of the estrous cycle. Superoxide dismutase 2 (SOD2) is known to play a crucial role as the major antioxidant defense system with increased expression under inflammatory conditions [30,31]. This enzyme efficiently converts superoxide to the less reactive hydrogen peroxide (H_2_O_2_), which can diffuse out of mitochondria and be further detoxified to water by other antioxidant enzymes [32]. It has been reported that the antioxidant system (including SOD2) plays an important role in the maintenance of CL integrity and function during the estrous/menstrual cycle [33]. The luteal expression of *SOD2* appears to be dependent on the stage of the estrous cycle as well as the activity of various immune cells [24]. 

To confirm our transcriptomic results, we performed biochemical analyses to determine antioxidant status. We found that LPS reduced the total antioxidant capacity of the CL and decreased the activity of key antioxidant enzymes such as catalase, superoxide dismutase, and glutathione-s-transferase. It should be noted that *SOD2* gene expression was upregulated after LPS treatment, whereas superoxide dismutase activity decreased. The lack of correlation between mRNA and protein expression has been frequently described and is the result of differences in mRNA and protein stability and the differential regulation of post-transcriptional and translational processes [34,35,36].

An interesting part of our present research is the identification of genes involved in the immune response, such as *TNFSF14*, *NLRP6*, *IL-6* and *BMX*. Of particular interest seems to be *TNFSF14* (TNF Superfamily Member 14), which was upregulated in the CL after the treatment with LPS. TNFSF14 is known to be a pro-inflammatory cytokine produced mainly by macrophages and T cells [37]. TNFSF14 has been shown to promote the activation and maturation of T lymphocytes [38] and increase the production of ROS [39], which subsequently leads to severe inflammation and tissue destruction. In addition, TNFSF14 has recently been proposed as one of the biomarkers for PCOS [40]. These results confirm that the use of LPS in the proposed experimental model induces an inflammatory response in porcine CL. 

In the present studies, we investigated the effect of PPARβ/δ ligands on the CL treated with LPS under in vitro conditions. It is worth noting that stimulation with LPS increased the expression of PPARβ/δ, suggesting its regulatory role in inflamed tissue. Our experimental model included two concentrations of PPARβ/δ selective agonist (GW0724)—1 μmol/L and 10 μmol/L. We found that 1 μmol/L of GW0724 affected the expression of 102 DEGs (63 DEGs were downregulated and 39 DEGs were upregulated), whereas 10 μmol/L of GW0724 altered the expression of 105 DEGs (58 DEGs were downregulated and 57 DEGs were upregulated). Most of these DEGs were involved in processes related to the regulation of oxidative stress and inflammation. Only the most interesting DEGs are discussed below. 

In this study, we demonstrated that the activation of PPARβ/δ by GW0724 affected the expression of genes related to the control of oxidative stress, such as *ALDH3B2*, *SURF1*, *DUOXA2* and *PDK4*. The treatment of the LPS-stimulated CL with PPARβ/δ agonist at both doses decreased the expression of *ALDH3B2* (described earlier in the discussion) and *SURF1* (Surfeit locus protein 1), which is involved in the proper assembly of cytochrome c oxidase (COX) [41]. It is worth noting that these genes were upregulated after treatment with LPS alone, suggesting that activation of PPARβ/δ reverses the negative effect of LPS. Our study also showed the downregulation of *DUOXA2* (maturation factor of DOUX2) after treatment with GW0724 at a concentration of only 1 μmol/L. DUOX2 is a membrane-localized glycoprotein composed of six transmembrane helices. In the presence of DUOXA2, these structural components regulate the transfer of electrons from NADPH to molecular oxygen to generate H_2_O_2_ [42]. DOUXA2 expression has been reported to be increased during chronic inflammation and in various cancers, which may be related to the extensive production of ROS [43,44]. DUOX2 upregulation has also been associated with a significant increase in extracellular H_2_O_2_ production and DNA damage in tissues [45]. In addition, it has been suggested that the pro-oxidant state resulting from the upregulation of DOUX2 may impede the recovery of tissue damage caused by inflammatory stress [44]. Moreover, the current study also demonstrated that blocking PPARβ/δ by an antagonist upregulated ENOX1 (Ecto-NOX disulfide thiol exchanger), a member of the ecto- NOX family involved in intracellular redox homeostasis [46]. ENOX1 has been reported to induce oxidative stress in human aortic endothelial cells [47]. Biochemical analyses determining antioxidant status confirmed the transcriptomic results. We demonstrated that the PPARβ/δ agonist reversed the LPS effect by increasing the activity of superoxide dismutase, glutathione transferase and catalase. The obtained results suggest that the use of the PPARβ/δ agonist attenuates oxidative stress and prevents tissue damage. Conversely, blocking the receptor may increase oxidative stress.

The present study has revealed the regulatory role of GW0724, a PPARβ/δ agonist, in the inflammatory process in the porcine CL. Interestingly, the observed effects appear to be dependent on the dose of ligand administered. The treatment with GW0724 at a concentration of 1 μmol/L revealed six DEGs (*CSF3*, *VTN*, *IL-15*, *C1QTNF12*, *DUOXA2*, *TNFSF14*) involved in the regulation of the inflammatory response or immune processes, according to the Gene Ontology analysis. In this work, we have demonstrated the inhibitory effect of GW0724 on the expression of CSF3 (Granulocyte colony-stimulating factor 3), the major regulator of neutrophil production [48]. CSF3 has been reported to exert pro-inflammatory properties in inflammatory joint diseases. There is also evidence that a deficiency of CSF3 protects mice from acute and chronic arthritis [48]. An inhibitory effect of GW0724 on the expression of a potent proinflammatory cytokine—*TNFSF14*—was also observed. It is worth noting that this is the opposite effect to that observed after LPS treatment alone.

The current results showed that GW0724 (1 μmol/L) decreased the expression of *VTN* (Vitronectin), a pro-inflammatory glycoprotein that binds to integrin receptors [49]. VNT-deficient mice were found to have lower numbers of neutrophils and lower concentrations of pro-inflammatory cytokines such as IL-1β and IL-6 in the lungs after LPS exposure than VTN-positive mice [50]. Moreover, the exposure of mice to VTN was associated with the decreased apoptosis of neutrophils [51]. In addition to its anti-apoptotic effect, VTN may also exacerbate the severity of acute lung injury by decreasing the uptake and clearance of apoptotic neutrophils by alveolar and tissue-derived macrophages, which is associated with the release of pro-inflammatory mediators [52].

The treatment with GW0724 (1 μmol/L) upregulated the expression of *IL-15* in inflamed CL. Interleukin 15 is a pleiotropic cytokine involved in the inflammatory response in various infectious diseases [53]. It has been reported that IL-15 plays an important role in host defense in sepsis induced in mice by *E. coli* [54]. Mice overexpressing IL-15 were resistant to the septic shock induced by *E. coli*, which was related to the inhibition of apoptosis triggered by TNF-α. Moreover, the treatment of normal mice with exogenous IL-15 made them resistant to *E. coli*-induced lethal shock [54].

The treatment of inflamed CL with GW0724 at a concentration of 10 μmol/L affected the expression of eight genes involved in the regulation of inflammatory responses or immune processes (*CD180*, *IL-6*, *CCL3L1*, *LTα*, *CCL4*, *ELF3*, *ZFP36*, *TNFSF14*). In contrast to the lower dose of GW0724 (1 μmol/L), which showed an anti-inflammatory character, the higher dose (1 μmol/L) seems to be pro-inflammatory. We have shown that the expression of *CD180*, a specific inhibitor of TLR4-mediated inflammatory response [55], was downregulated after the treatment of inflamed CL with GW0724 at the higher dose. CD180 is an accessory TLR4 molecule expressed in various cell types, including monocytes and macrophages [30]. In addition, we detected the increased expression of *IL-6*, *CCL3L1*, *CCL4*, *LTα* and *ELF3*, which are genes known to possess pro-inflammatory properties, mainly expressed through the induction of chemotaxis and the activation of lymphocytes and macrophages [56,57,58,59].

Statistical analysis performed between the two PPARβ/δ agonist doses revealed 19 downregulated and 77 upregulated genes. The most interesting genes are involved in the regulation of inflammatory and immune responses. Among them are *MBL1*, *CCL19*, *IL-17β*, *PGLYRP3* and *CSF3*, whose expression was higher after treatment with GW0724 at a concentration of 10 μmol/L compared with 1 μmol/L. Mannan-binding lectin (MBL) is an important factor of innate immunity that contributes to the elimination of microorganisms. MBL has been reported to bind to bacteria and then neutralize them by opsonizing and activating complement through the lectin pathway of complement activation [60]. In turn, peptidoglycan recognition protein 3 (PGLYRP3) recognizes bacterial compounds (peptidoglycan) and plays a role in antibacterial innate immunity [61]. Both factors are crucial during the first step of bacterial infection. The chemokine CCL19 triggers T cell proliferation, leading to upregulation of pro-inflammatory cytokine synthesis [62]. It has been reported that IL-17B induces monocytes to produce TNF-α and IL-1β and supports neutrophil recruitment and B cell chemotaxis [63,64]. During infection, immune cells such as granulocytes, macrophages, and lymphocytes are recruited to tissues to clear bacterial infection [6]. We propose that PPARβ/δ may not only play a key role in alleviating chronic inflammation, but may also be helpful in supporting the immune response to bacterial infection in the CL. 

## 4. Materials and Methods

### 4.1. Experimental Animals

The study was conducted on corpora lutea harvested from gilts intended for commercial slaughter and meat processing in accordance with the guidelines for animal care (the Act of 15 January 2015 on the Protection of Animals Used for Scientific or Educational Purposes and Directive 2010/63/EU of the European Parliament and the Council of 22 September 2010 on the protection of animals used for scientific purposes). Experimental material was collected from adult crossbred gilts (Large White × Polish Landrace, 7 months old, 100 kg body weight, *n* = 4) on days 10–12 of the estrous cycle (mid-luteal phase). On the farm, pigs were observed in two consecutive heat cycles. The first mark of estrus (the behavior of gilts observed in the presence of the boar) was defined as day 0 of the estrous cycle. The animals were transported to the local slaughterhouse where the ovaries were dissected within a few minutes. The removed tissues were transferred to the laboratory on ice in phosphate-buffered saline (PBS) with an antibiotic cocktail (100 IU/mL penicillin and 100 mg/mL streptomycin, PolfaTarchomin, Poland). The phase of the estrous cycle was proven in the laboratory from the morphological characteristics of the ovary [65].

### 4.2. In Vitro Experiment

The procedure for collection and incubation of the porcine CL was previously described [66]. In the laboratory, the CL were dissected from the ovary, connective tissue was removed, and placed on ice in a sterile Petri dish. CLs were cut into small pieces (100 ± 10 mg, in duplicate from each experimental replicate). Each tissue explant was placed in M199 medium (Sigma Aldrich, St. Louis, MO, USA) supplemented with 0.1% BSA fraction V (Roth, Germany) and antibiotics. The explants were pre-incubated for 2 h in a water bath at 37 °C in an atmosphere of 95% O_2_ and 5% CO_2_. Then, the explants were treated with LPS (100 ng/mL, from *E. coli*) for 24 h. Explants not treated with LPS were considered as controls. The medium was removed, and the explants were incubated for 6 h with LPS alone or in combination with the PPAR β/δ ligands: GW0724 (agonist; 1 μmol/L or 10 μmol/L, Cayman Chemical Company, Ann Arbor, MI, USA) or GSK3787 (antagonist; 25 μmol/L, Cayman Chemical Company). Controls also contained dimethyl sulfoxide (DMSO, solvent for the tested PPAR ligands). After the incubation, tissue explants were frozen at −80 °C until further analysis.

### 4.3. RNA Isolation, Library Preparation and Sequencing Procedure

Total RNA from 20 samples was isolated using the “RNeasy Mini Kit” (Qiagen, Hilden, Germany) according to the manufacturer’s protocol. The Tecan Infinite M200 plate reader (Tecan Group Ltd., Männedorf, Switzerland) and Agilent Bioanalyzer 2100 (Agilent Technology, Santa Clara, CA, USA) were used to evaluate total RNA quantity and quality. The samples with an RNA Integrity Number (RIN) of >7 were selected for the next analyses. The poly(A) RNA-sequencing library was prepared according to the Illumina TruSeq Stranded mRNA Sample Preparation Protocol. Two rounds of purification were performed using oligo(dT) magnetic beads to purify the poly(A) tailed mRNA. Subsequently, the poly(A) RNA was fragmented at high temperature using a divalent cation buffer, and poly(dT) oligonucleotides were used to transcribe the RNA into cDNA. Subsequently, the cDNA was subjected to 3’ tail adenylation and adapter ligation. Reverse transcription during library construction was strand-specific. Finally, the libraries were pooled and then sequenced. Quality control analysis and quantification of the sequencing libraries were performed using the Agilent Technologies 2100 Bioanalyzer High Sensitivity DNA Chip. Paired-end sequencing was performed using the Illumina NovaSeq 6000 Sequencing System (LC Science, Houston, TX, USA).

### 4.4. Transcript Assembly and Analysis of Differentially Expressed Genes

FastQC was used to assess sequence quality. After removing low-quality reads, the remaining 150 bp paired-end sequences were reassembled and mapped to the *Sus scrofa* genome using HISAT2 [67,68]. The mapped reads from each sample were assembled using StringTie [68]. All transcriptomes were then merged to reconstruct a comprehensive transcriptome using Perl scripts and GffCompare. Once the final transcriptome was constructed, StringTie and edgeR [69] were used to estimate the expression levels of all transcripts. StringTie was used to determine the expression of mRNAs by calculating fragments per kilobase of transcript per million (FPKM) [68]. Differentially expressed genes (DEGs) were selected with log_2_ (fold change) > 1 or log_2_ (fold change) < −1 and with statistical significance (*p*-value < 0.05) using the R package edgeR [69].

### 4.5. Real-Time PCR

Differentially expressed genes were validated via real-time PCR using the AriaMx real-time PCR System (Agilent Technology, Santa Clara, CA, USA), as previously described [70]. Primer sequences (Supplemental Table S1) for reference and target genes (*IL-6*, *SOD2*, *CD180*, *ANGTPL4*, *PPARβ/δ*) were designed via Primer Express Software 3 (Applied Biosystems, Waltham, MA, USA). The abundance of the tested mRNAs was calculated using the comparative Pfaffl method [71]. The constitutively expressed *ACTB* and *GAPDH* genes were implemented as reference genes, and the geometric mean values of the expression levels were used for analysis. Real-time PCR results were analyzed using Statistica software (version 13.1; Statsoft Inc. Tulsa, OK, USA) with Student’s *t* test and expressed as means ± SEM. Results were considered statistically significant at *p* ≤ 0.05.

### 4.6. Biochemical Analyses

#### 4.6.1. Tissue Extract Preparation for Biochemical Analyses

The extract of the CL tissue after in vitro culture for biochemical analyses (in 5 technical replicates of each sample) was prepared via mechanical homogenization (Omni tissue Homogenizer, Kennesaw, GA, USA) in sterile PBS. Extracts were centrifuged (5000× *g*) at 4 °C for 15 min, and the supernatant was transferred to new tubes containing 500 μL. Protein concentration was determined using the bicinchoninic acid method (Pierce BCA Protein Assay Kit, Thermo Fisher Scientific, Waltham, MA, USA) according to the manufacturer’s protocol.

#### 4.6.2. Antioxidant Capacity

Total antioxidant capacity (TAC) was analyzed using the improved ABTS radical cation decolorization assay according to Re et al. [72]. The pre-formed radical monocation of 2,2’-azinobis-(3-ethylbenzothiazoline-6-sulfonic acid) (ABTS*^+^) was generated via the oxidation of ABTS with potassium persulfate and was reduced in the presence of such hydrogen-donating antioxidants. The results were calculated as Trolox (a water-soluble analogue of vitamin E) equivalents per L per mg of protein. 

#### 4.6.3. Peroxidase Activity

Preoxidase activity was determined according to the method described by Chance and Maehly [73]. The method consists of determining the content of purpurogallin, an orange crystalline compound in the incubation mixture, formed when pyrogallol is oxidized as a hydrogen donor in the presence of hydrogen peroxide. Samples were mixed with pyrogallol and hydrogen peroxide and incubated at 30 °C for 4 min. Absorbance was measured at 430 nm against air. The difference between the absorbance of the control sample (0.05 M acetate buffer at pH 5.6 was added instead of the tissue homogenate) and the tested sample (tissue homogenate) was a measure of enzyme activity. The millimolar absorbance coefficient for purpurogallin was 2.47/mM·cm. Enzyme activity was converted to mg of protein in the assay.

#### 4.6.4. Catalase Activity

The measurement method is based on the ability of catalase to decompose hydrogen peroxide [74]. The reaction is accompanied by a decrease in absorbance at a wavelength of 240 nm. Briefly, samples were diluted 20 times with 0.2 M phosphate buffer at pH 7. A total of 100 µL of H_2_O_2_ was then added to 200 µL of the sample. The absorbance was measured relative to the control (buffer instead of sample) for 30 s at 5 s intervals. The value of the decrease in absorbance was determined and the activity expressed in katal per mg of protein.

#### 4.6.5. Superoxide Dismutase Activity

The method for determining the activity of superoxide dismutase (SOD) uses the ability of p-iodonitrotetrazolium [2-(4-iodophenyl-3-(4-nitrophenyl)-5-phenyltetrazolium; INT] to be reduced to a water-soluble product with an absorption maximum at about 505 nm (reddish pink) by superoxide anion (O^2−^), which is formed during the oxidation reaction of xanthine by xanthine oxidase [74]. The rate of reduction of INT is linearly related to the activity of xanthine oxidase and is inhibited by SOD. Superoxide dismutase inhibits the reduction of INT to purple formazan by scavenging this radical. The rate of formazan formation is a measure of the activity of SOD [75]. The activity of SOD was expressed in arbitrary units [a. u.] per mg of protein.

#### 4.6.6. Glutathione S-Transferase Activity

The glutathione S-transferase (GST) activity was determined using the Rice-Evans [76] method. Enzyme activity was calculated based on the millimolar absorption coefficient (9.6 mmol^−1^/cm^−1^) for the glutathione conjugate formed from 1-chloro-2,4-dinitrobenzene. The GST activity was converted to arbitrary units [a. u.] per mg of protein. 

#### 4.6.7. Statistical Analysis for Biochemical Analyses

Statistical analysis for the obtained results was performed using *t*-test in Prism 9 software (version 9.1.1 (223); GraphPad Software Inc., San Diego, CA, USA). Results were considered statistically significant at *p* ≤ 0.05 (*) and *p* ≤ 0.002 (**).

## 5. Conclusions

In conclusion, this is the first report describing the in vitro effects of different doses of PPARβ/δ agonist (GW0742) on LPS-induced inflammation in the CL. We imply that PPARβ/δ ligands act in two ways depending on the dose. We have shown that both doses of the ligand exert a positive effect on the oxidative status during inflammation. Moreover, we postulate that lower dose of GW0724 effectively inhibits the expression of potent pro-inflammatory mediators, whereas the higher dose increases the expression of pro-inflammatory factors, which are mostly responsible for the induction of chemotaxis and the functional and proliferative activation of leukocytes. Therefore, we propose that the lower dose of GW0724 can be used to alleviate chronic inflammation, while the higher dose can be used to support the natural anti-pathogen response during the acute phase of inflammation that occurs at the onset of bacterial infection.

## Data Availability

The datasets generated and/or analyzed during the current study are available in the ArrayExpress database; Accession: E-MTAB-12027 https://www.ebi.ac.uk/biostudies/arrayexpress/studies/E-MTAB-12027 (accessed on 1 September 2022).

## Supplementary Materials

The following supporting information can be downloaded at: https://www.mdpi.com/article/10.3390/ijms24054993/s1.
