# Supplementary material for: PPARβ/δ Ligands Regulate Oxidative Status and Inflammatory Response in Inflamed Corpus Luteum—An In Vitro Study
Source: Int J Mol Sci. 2023 Mar 5;24(5):4993. doi: 10.3390/ijms24054993 (PMC10003567; doi:10.3390/ijms24054993)
Supplement: Supplementary file 1 [file ijms-24-04993-s001.zip › Supplemental figures.pdf]

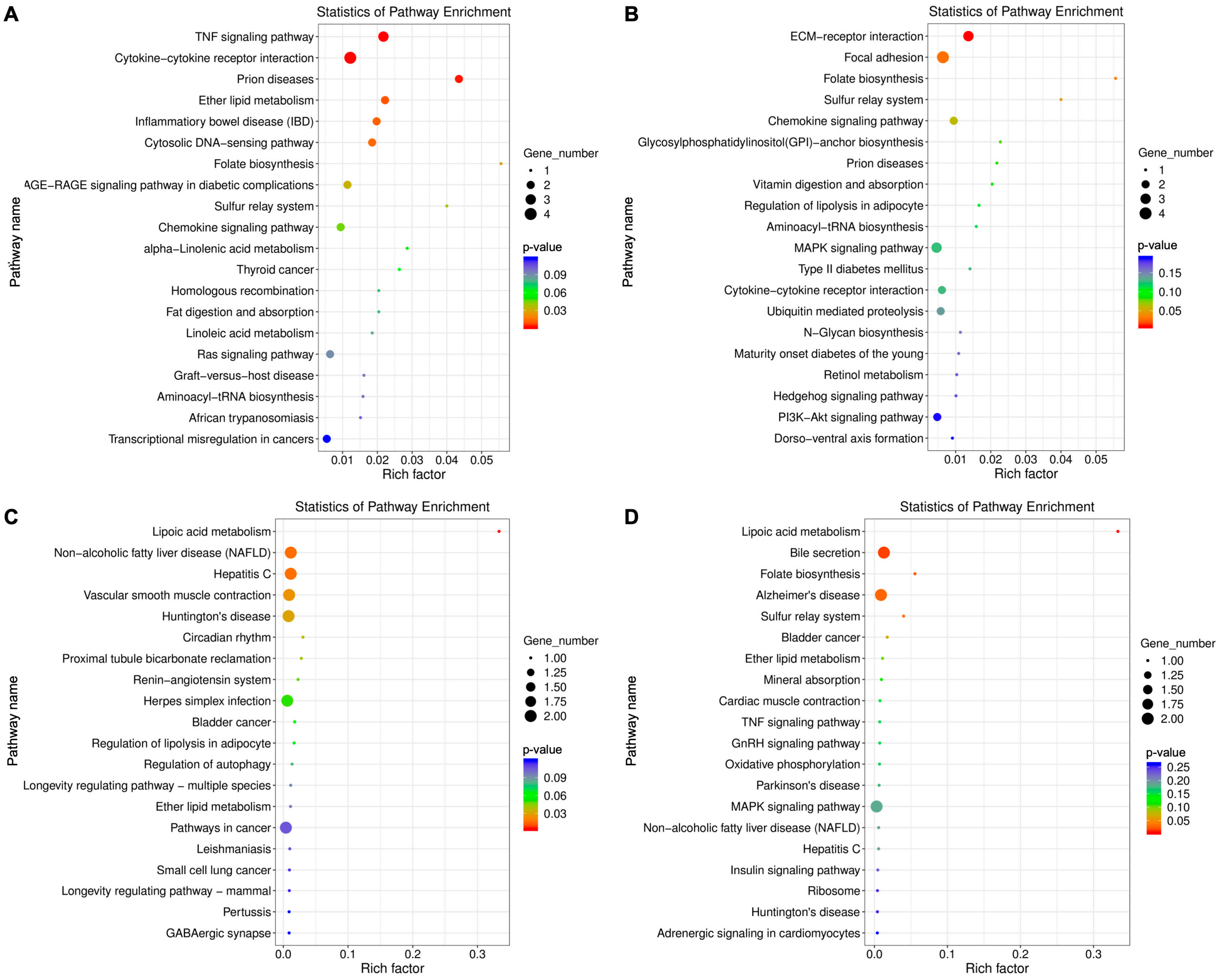

Figure S1: KEEG analysis of differentially expressed genes in the corpus luteum after LPS (A); treatment or LPS in combination with: GW0724 at a concentration of 1 µmol/L (B); GW0724 at a concentration of 10 µmol/L (C); GSK3787 treatment (D). The color indicates the *p* value of each pathway, while the size of the circle represents gene number in each pathway.

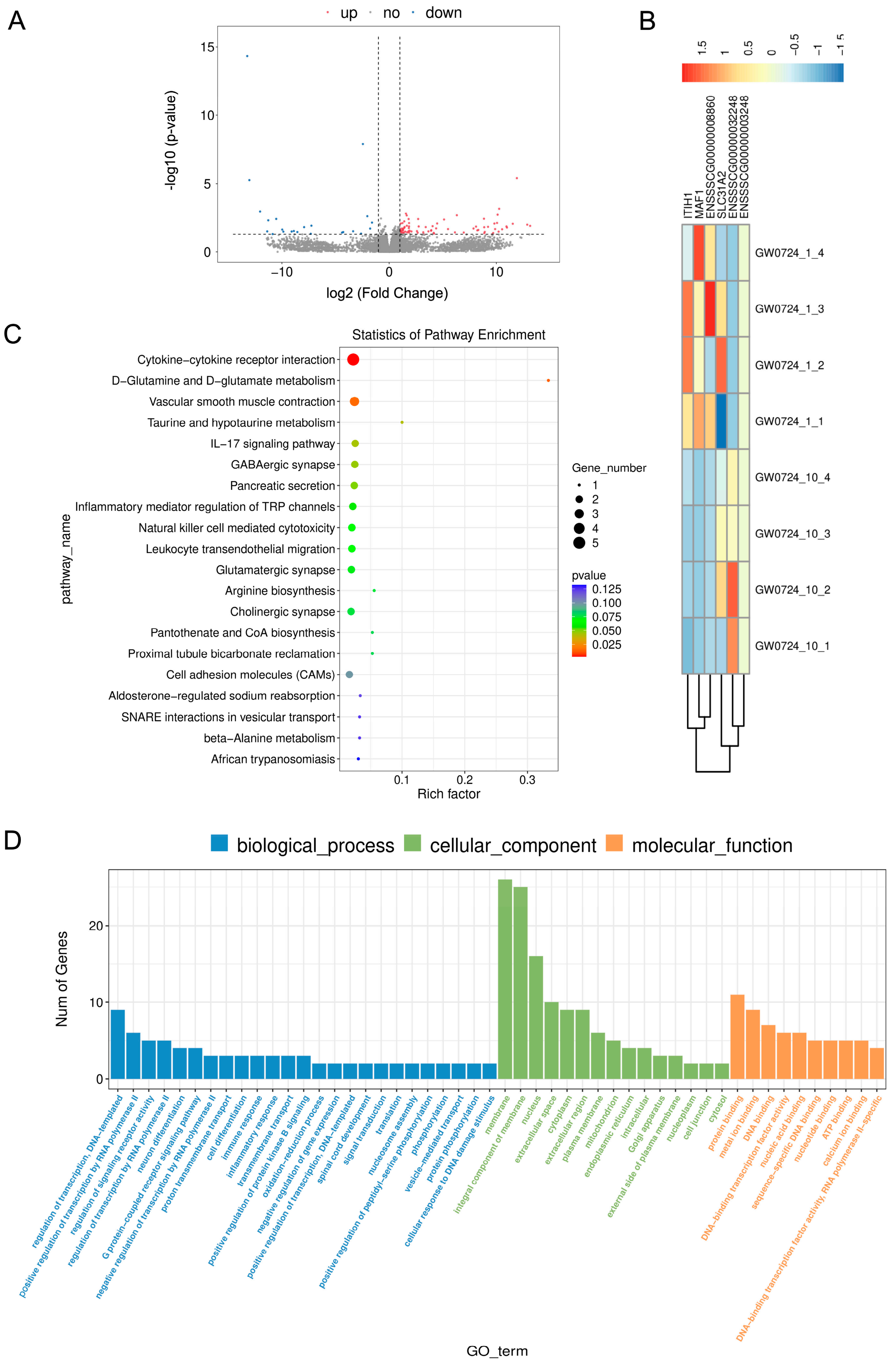

Figure S2: Volcano plots describing the abundance of transcript expression profiles between GW0724 at a concentration of 1 μmol/L and GW0724 at a concentration of 10 μmol/L (A); hierarchical clustering heatmap of differentially expressed genes between GW0724 at a concentration of 1 μmol/L and GW0724 at a concentration of 10 μmol/L (B); KEGG analysis of the differentially expressed genes between GW0724 at a concentration of 1 μmol/L and GW0724 at a concentration of 10 μmol/L (C); GO analysis of the differentially expressed genes between GW0724 at a concentration of 1 μmol/L and GW0724 at a concentration of 10 μmol/L (D).

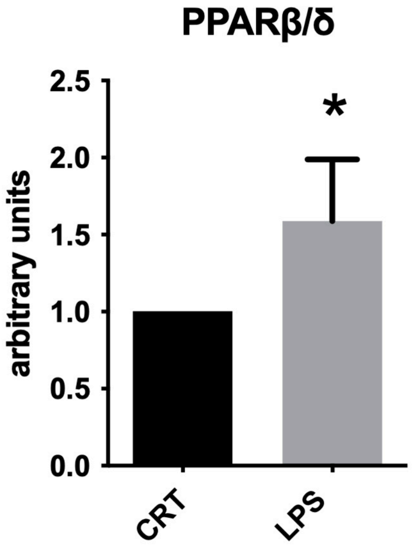

Figure S3: Expression of *PPARβ/δ* mRNA in porcine corpus luteum during mid-luteal phase after LPS treatment. Results were considered statistically significant at $p \leq 0.05$ (*).

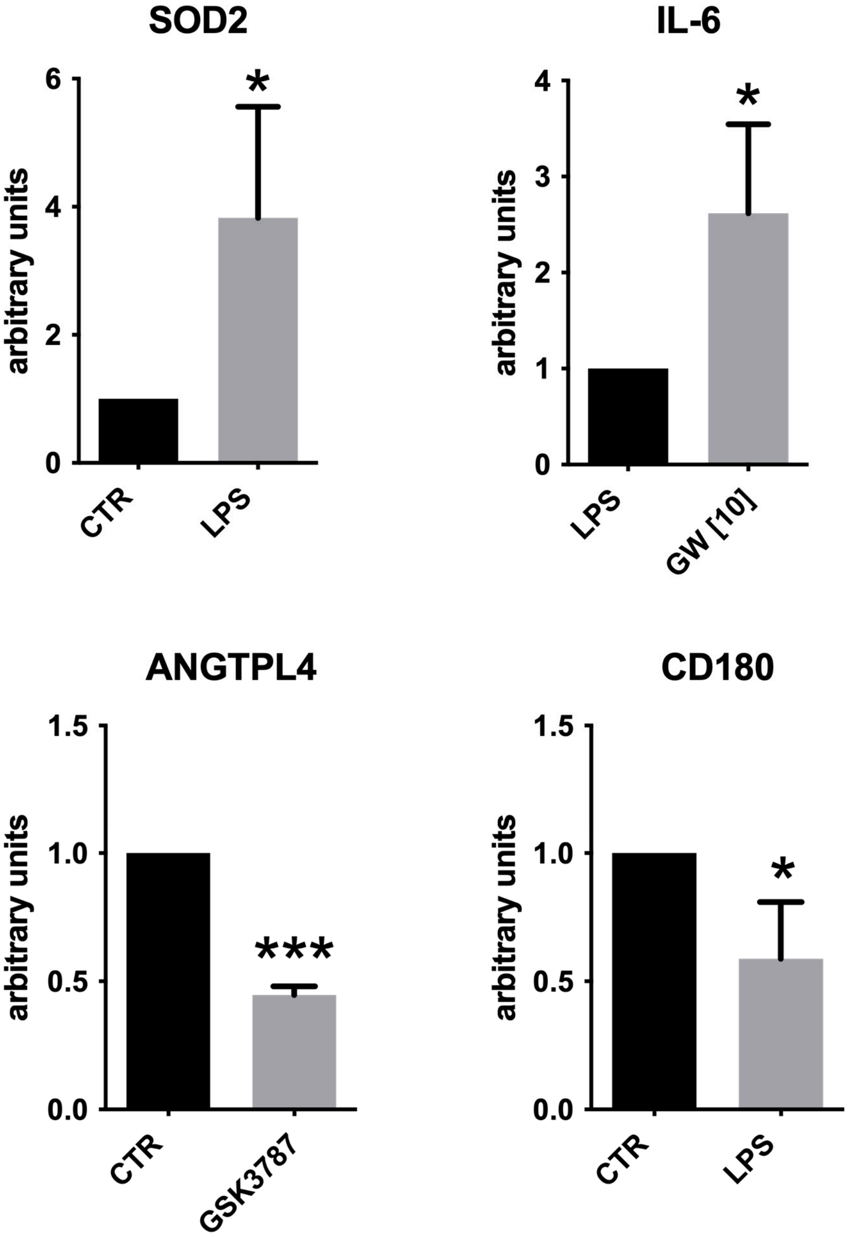

Figure S4: Real-time PCR validation of RNA-Seq results for random differentially expressed genes. Results were considered statistically significant at $p \leq 0.05$ (*), and $p \leq 0.001$ (***).
